# Unbiased Long-Read Whole-Genome Sequencing Enables High-Resolution Mapping of Transgene Concatenation and Off-target Genomic Disruption in a Mouse Model

**DOI:** 10.34133/csbj.0234

**Published:** 2026-10-08

**Authors:** Maanik Mehta, Kashif Ahmed, Rowaida Hussein, Erika Tavares, Zorana Berberovic, Rabiat Adele, Abigail D’Souza, Bin Gu, Michael D. Wilson, Evgueni Ivakine, Philippe P. Monnier, Elise Héon, Ajoy Vincent

**Affiliations:** ^1^ Genetics and Genome Biology Program, Hospital for Sick Children, Toronto, ON M5G 0A4, Canada.; ^2^ Institute of Medical Science, University of Toronto, Toronto, ON M5S 1A8, Canada.; ^3^ The Centre for Phenogenomics, Toronto, ON M5T 3H7, Canada.; ^4^ Lunenfeld–Tanenbaum Research Institute, Mount Sinai Hospital, Toronto, ON M5G 1X5, Canada.; ^5^ Department of Obstetrics, Gynecology and Reproductive Biology, College of Human Medicine, Michigan State University, East Lansing, MI 48824, USA.; ^6^ Institute for Quantitative Health Science and Engineering, Michigan State University, East Lansing, MI 48824, USA.; ^7^ Department of Molecular Genetics, Faculty of Medicine, University of Toronto, Toronto, ON M5S 3K3, Canada.; ^8^ Department of Physiology, Faculty of Medicine, University of Toronto, Toronto, ON M5S 3K3, Canada.; ^9^ Krembil Research Institute, Toronto Western Hospital, Toronto, ON M5T 0S8, Canada.; ^10^ Department of Ophthalmology and Vision Sciences, Hospital for Sick Children and University of Toronto, Toronto, ON M5T 3A9, Canada.

## Abstract

Transgenic mouse models are indispensable for dissecting disease mechanisms, yet their interpretability is frequently compromised by cryptic genomic alterations introduced during transgenesis. Thus, robust quality-control strategies are needed to elucidate integration architecture and evaluate model performance when such unintended events occur. Here, we applied unbiased whole-genome long-read sequencing using the PacBio Revio to investigate a mouse model exhibiting unexpected transgene silencing, originally designed to recapitulate autosomal-dominant hereditary macular dystrophy driven by the up-regulation of a *ZZEF1-ALOX15* fusion gene. Long-read sequencing analysis revealed a ≥29-kb head-to-tail concatemer containing at least 3 transgene-derived copies. Reconstruction of transgene–genome junctions revealed unintended off-target integration of the concatemer into the calcium-sensing receptor gene (*Casr*), along with exogenous *Escherichia coli* DNA, that together defined final transgene architecture. Aggregate 5-methylcytosine profiling identified hypermethylation of the transgene promoter across the concatemer, and additional phenotyping suggested disruption of endogenous CaSR function resulting from the rearrangement. Our workflow enabled direct detection of transgene concatenation and off-target mapping. These findings highlight the power of whole-genome long-read sequencing in uncovering hidden genomic complexity, resolving aberrant phenotypes, and enhancing the reliability of in vivo disease modeling, supporting its incorporation as a potentially scalable quality-control tool for genetically engineered animal models.

## Graphical Abstract

**Figure:**
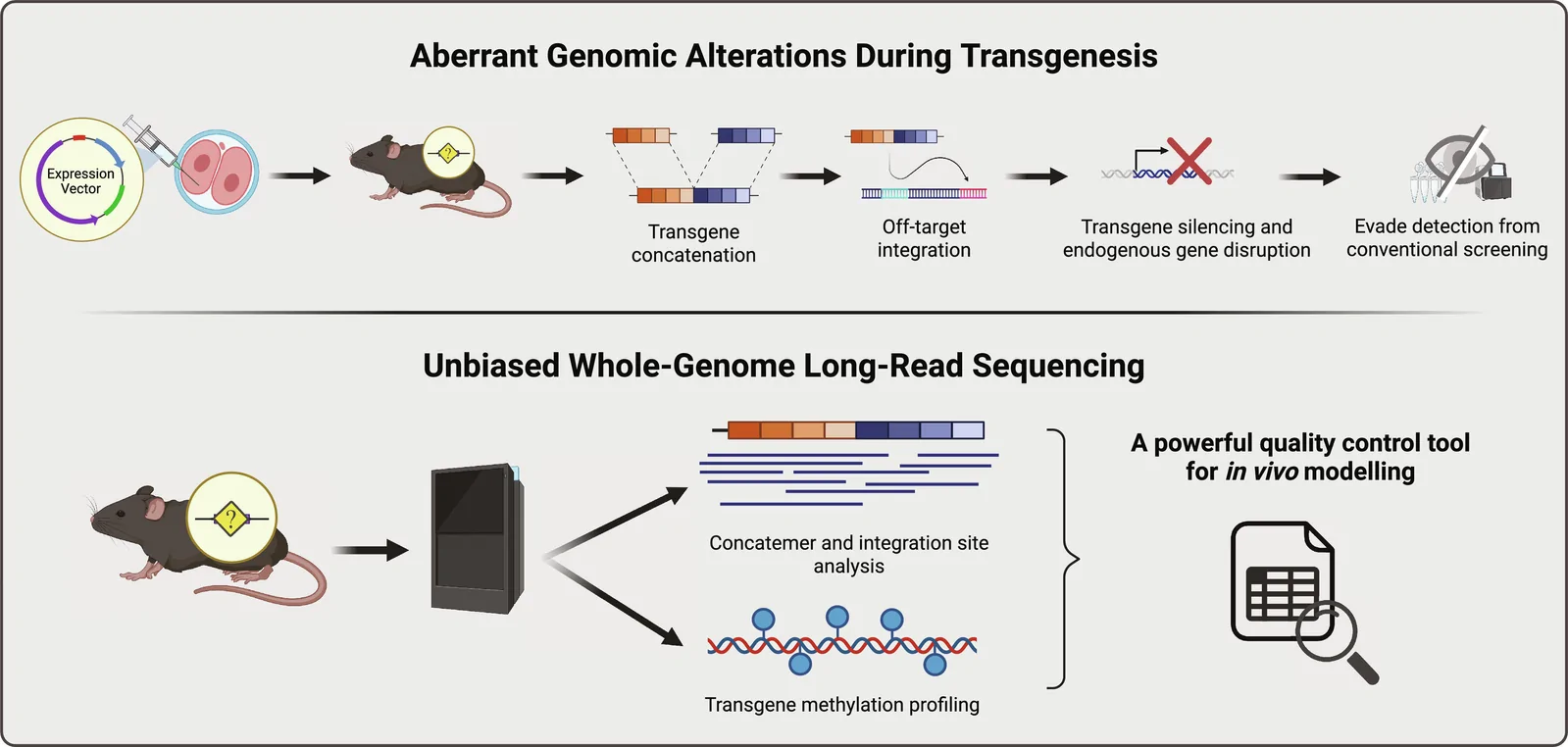

## Introduction

The development of genetically engineered mice over the past decade has heralded our unprecedented ability to interrogate the complex mechanisms of human disease. Today, a suite of powerful editing tools can enable precise, “on-target” manipulations of the murine genome, ranging from single-base-pair modifications to multi-kilobase insertions, deletions, and duplications [1–4]. While the CRISPR-Cas9 system remains the preferred gene-editing strategy, its efficiency in facilitating large on-target insertions is limited [5]. Moreover, unintended rearrangements between transgenes and the host genomes are frequently observed during transgenesis and can confound phenotypic characterization by disrupting endogenous gene function [6–9]. These include the concatenation of transgenic DNA during cell microinjection, whereby copies of plasmid recombine into tandem arrays prior to genomic integration [10,11]. Identification of concatemers is critical to deciphering model viability, as their formation can result in aberrant transgene copy number, loss of plasmid elements, or even repeat-induced gene silencing (RIGS) [10,12,13]. Nonetheless, these unintended outcomes may be missed by traditional polymerase chain reaction (PCR)-based methods due to technical limitations in amplifying large and/or repetitive transgenes, the loss of primer binding sites, multiple integration sites, or preservation of integration junctions [11,14–16]. Conventional short-read next-generation-sequencing technologies, which can generally map integration sites through split/discordant read analysis, similarly struggle to characterize repetitive transgene rearrangements when these structures exceed the read length yields of 150 bp that most platforms provide, even with sequencing coverage as high as 250× [17–20]. Thus, robust quality-control measures that permit reliable and efficient detection of such effects are needed.

In recent years, long-read sequencing (LRS) platforms such as Pacific Biosciences (PacBio) or Oxford Nanopore Technologies (ONT) have emerged as novel methods to elucidate transgene architecture. The primary benefit of LRS is its ability to generate median read lengths of 10 to 100 kb, capturing repetitive DNA and concatemer–host genome junctions within a single read [21,22]. An added advantage for both PacBio and ONT over short-read sequencing is their ability to perform onboard methylation profiling, concurrently informing transgene structure and its expression potential [23–25]. Early transgene mapping attempts using whole-genome LRS yielded only a minute fraction of reads capturing the transgene–genome junctions, demonstrating considerable inefficiencies with these sequencing workflows [26,27]. Accordingly, LRS is frequently combined with Cas9-based enrichment strategies, which selectively cleave DNA within the transgene or around a predetermined locus for adaptor ligation and sequencing [28]. While these techniques achieve exceptional coverage of the integration locus, their targeted nature offers limited utility in examining unwanted editing outcomes that are not physically linked to the captured transgene sequence [9,29,30]. Thus, when the underlying mechanism of model failure or inefficiency is unknown, a comprehensive genome-wide strategy offering nontargeted resolution would be particularly advantageous [31].

Here, we describe the application of unbiased whole-genome LRS to investigate transgene silencing in a mouse model, designed to recapitulate a form of autosomal-dominant hereditary macular dystrophy driven by the ectopic overexpression of a 3.3-kb *ZZEF1-ALOX15* fusion gene [32]. Previous in vivo electroporation of the *ZZEF1-ALOX15* chimera demonstrated retinal photoreceptor damage, motivating the generation of a stable mouse line for mechanistic studies [32]. Thus, a Cre-inducible expression vector (*pCAG-ZZEF1-ALOX15-T2A-L2T*) containing the chimera was targeted to the *ROSA26* safe harbor locus in mice using CRISPR-Cas9. The generated line was subsequently crossed to retina-specific Cre-driver strains. Despite passing standard quality control at the generation facility, the resulting mice displayed no evidence of transgene activation, prompting deeper investigation. We performed PacBio high-fidelity (HiFi) sequencing of the mouse model, identifying a complex multicopy transgene concatemer with unintended off-target integration into the calcium-sensing receptor gene (*Casr*) and hypermethylation of the transgene promoter in ear-notch DNA. Systemic evaluation revealed altered calcium homeostasis in transgenic mice as a functional consequence of the rearrangement. Together, these findings provide a mechanistic framework for the failure of transgene activation in this model and demonstrate the broad utility of LRS as a quality-control tool for the characterization of genetically engineered mice.

## Materials and Methods

### Mouse housing and maintenance

Mice were housed at The Centre for Phenogenomics (TCP, Toronto, Canada) in accordance with the Canadian Council on Animal Care guidelines and mandates established by Ontario’s Animals for Research Act. Animals were provided ad libitum access to water and standard chow and were maintained under aseptic conditions with a 12-h light/dark cycle. All animal protocols were approved by the TCP Animal Care Committee (AUP 29-0368).

### Generation of transgenic mouse model

A 19-kb construct (*pCAG-ZZEF1-ALOX15-T2A-L2T*) containing the 3.3-kb *ZZEF1-ALOX15* fusion gene and a Luciferase-tdTomato (L2T) fusion reporter, under control of the cytomegalovirus (CMV) early enhancer/chicken β-actin (CAG) promoter, was generated using InFusion cloning (Fig. 1A). A loxP-flanked stop cassette (LSL) immediately downstream of the CAG promoter was used to enable Cre-inducible activation of the transgene. The backbone plasmid contained an ampicillin resistance cassette as well as 5′ and 3′ homology arms (800 bp and 4.3 kb, respectively) to direct integration of the construct into the *ROSA26* locus. Of the 19-kb construct, the cassette for genomic integration (excluding backbone elements and homology arms) spanned 11 kb. The final construct (Data File S1) was validated by Sanger sequencing at The Centre for Applied Genomics (TCAG, The Hospital for Sick Children, Toronto, Canada). Construct functionality was assessed by co-transfection with pCMV-Cre (Addgene plasmid #123133) in HEK293T cells using Lipofectamine 3000 (L3000001; Invitrogen, Carlsbad, CA, USA), followed by imaging to detect tdTomato fluorescence 24 h post-transfection.

**Fig. 1.**
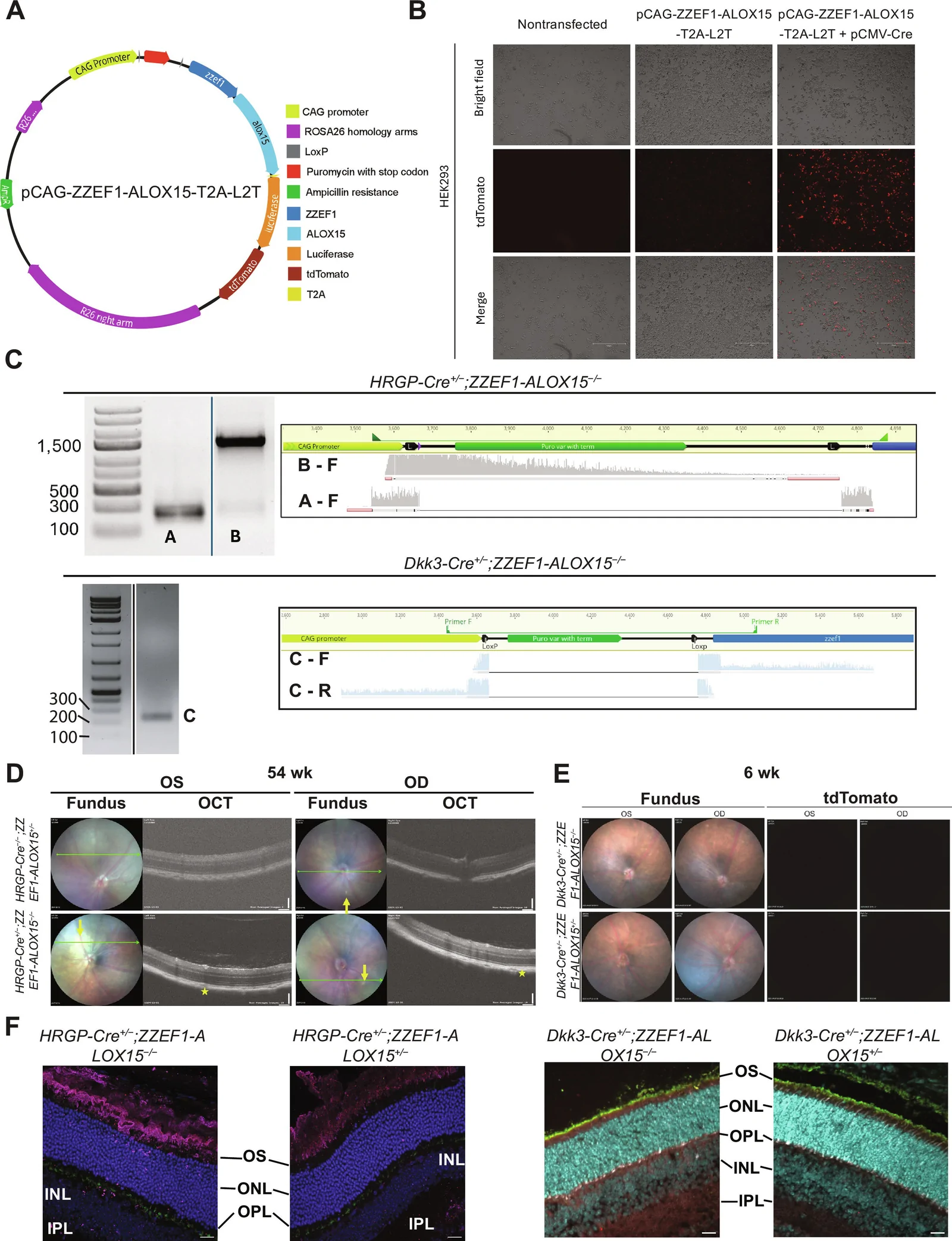
(A) Schematic of pCAG-ZZEF1-ALOX15-T2A-L2T designed to express the *ZZEF1-ALOX15* fusion gene, luciferase, and tdTomato under the control of the CAG promoter. (B) In vitro validation of the construct. Co-transfection of HEK293T cells with pCMV-Cre activated tdTomato reporter expression, confirming construct functionality. Images were captured at 10× original magnification; scale bars, 750 μm. (C) Gel electrophoresis and Sanger sequencing alignment (Geneious Prime) following PCR genotyping of retinal DNA from *HRGP-Cre^+/−^;ZZEF1-ALOX15^+/−^* and *Dkk3-Cre^+/−^;ZZEF1-ALOX15^+/−^* mice, showing excision of the loxP-flanked stop cassette. F, sequencing chromatogram from the forward primer; R, sequencing chromatogram from the reverse primer (Table S2). (D) Top panel: fundus and OCT examination of both eyes of an *HRGP-Cre^+/−^;ZZEF1-ALOX15^−/−^* littermate control at week 54. The small yellow spot in the right eye fundus is suggestive of drusenoid lesion development. Bottom panel: both eyes of an *HRGP-Cre^+/−^;ZZEF1-ALOX15^+/−^* mouse showing dots on fundus corresponding to hyper-reflective drusenoid lesions (*) at the retinal pigment epithelium. Retinal lamination appears preserved across both transgenic and control mice. (E) Fundus imaging of both eyes at week 5 showing a littermate control (*Dkk3-Cre^+/−^;ZZEF1-ALOX15^−/−^*) in the top panel and a transgenic mouse (*Dkk3-Cre^+/−^;ZZEF1-ALOX15^+/−^*) in the bottom panel. Both eyes appear unremarkable on examination. Imaging with a tdTomato excitation filter over the same region reveals no detectable fluorescence. (F) Cryo-immunohistochemistry showing absence of tdTomato reporter in both transgenic lines. In *HRGP-Cre^+/−^;ZZEF1–ALOX15^+/−^* sections (8 wk), nuclei were stained with 4′,6-diamidino-2-phenylindole (DAPI; cyan), rods with rhodopsin (purple), cones with cone arrestin (green), and tdTomato (red). In *Dkk3-Cre^+/−^;ZZEF1–ALOX15^+/−^* sections (11 d), nuclei were stained with DAPI (cyan), rods with rhodopsin (green), cones with cone arrestin (white), and tdTomato (red). Images were captured at 40× original magnification; scale bars, 20 μm. OS (panels D/E), left eye; OD, right eye; OCT, optical coherence tomography; OS (panel F), outer segment; ONL, outer nuclear layer; OPL, outer plexiform layer; INL, inner nuclear layer; IPL, inner plexiform layer.

An editing mix containing recombinant Cas9 protein, a guide RNA targeting the *ROSA26* locus (Table S1), and the donor plasmid was injected into C57BL/6J embryos at the 2-cell stage at the McGill Integrated Core for Animal Modeling (MICAM, McGill University, Montreal, Canada).

Resulting B6.Gt(ROSA)26Sor^tm1(CAG-ZZEF1/ALOX15)1Vin^ founder mice, referred to as *ZZEF1-ALOX15* mice hereinafter, underwent routine PCR-based quality control by MICAM, using primers spanning the 5′ and 3′ junctions to verify construct integration (Table S2). The founder line that passed this validation was then rederived at the TCP and bred to establish stable lines. PCR genotyping of the tdTomato reporter confirmed successful germline transmission (Table S2). Offspring from this line were then crossed by our group to the *HRGP-Cre* driver strain [33], obtained from the Jackson Laboratory (Bar Harbor, ME, USA; strain #032911), to selectively induce transgene activation in the cone-photoreceptor cell population. To enhance the probability of detecting transgene activation, transgenic mice were also crossed to the *Dkk3-Cre* line [34] (generated by Dr Takahisa Furukawa, University of Osaka, Osaka, Japan), which mediates expression in almost all retinal progenitor cells.

### Retinal phenotyping of Cre-activated litters

Retinae from transgenic mice carrying the *ZZEF1-ALOX15* construct and either the cone-photoreceptor-specific *HRGP-Cre* (*HRGP-Cre^+/−^;ZZEF1-ALOX15^+/−^*) or the pan-retinal *Dkk3-Cre* (*Dkk3-Cre^+/−^;ZZEF1-ALOX15^+/−^*) driver alleles were harvested at weeks 28 and 77, respectively, for DNA extraction. PCR from these extracts was performed using AmpliTaq Gold 360 Master Mix (Applied Biosystems; 4398881) to verify Cre-mediated excision of the LSL cassette in our construct (Table S2). Bands corresponding to the expected amplicon size were gel-extracted and re-amplified prior to Sanger sequencing.

Transgenic mice (*HRGP-Cre^+/−^;ZZEF1-ALOX15^+/−^* and *Dkk3-Cre^+/−^;ZZEF1-ALOX15^+/−^*) underwent descriptive ocular phenotyping alongside littermate controls. During in vivo imaging, mice were anesthetized using ketamine (100 mg/kg) and xylazine (10 mg/kg), which were administered intraperitoneally (0.10 mL/10 g body weight). Assessments included fundus photography and optical coherence tomography (OCT) using the Phoenix MICRON 5 camera (Phoenix Research Laboratories, Pleasanton, CA, USA) at months 1, 2, 3, 6, 9, and 12 for the *HRGP-Cre* x *ZZEF1-ALOX15* cohort [*HRGP-Cre^+/−^;ZZEF1-ALOX15^+/−^* (*n* = 4); *HRGP-Cre^+/−^*;*ZZEF1-ALOX15^−/−^* (*n* = 2)]. OCT examination provides high-resolution cross-sectional imaging of the retina, allowing detection of morphological changes to the retinal layers as those previously observed in human subjects [32]. Additionally, live fundus imaging with a tdTomato excitation/emission filter set (Phoenix MICRON 5) was performed on the *Dkk3-Cre* cohort at week 5 (*n* = 1, *Dkk3-Cre^+/−^;ZZEF1-ALOX15^+/−^*; *n* = 1, *Dkk3-Cre^+/−^;ZZEF1-ALOX15^−/−^*). For ex vivo evaluation, the retina and retinal pigment epithelium tissue were collected from a transgenic mouse and control littermate from each line (*Dkk3-Cre^+/−^;ZZEF1-ALOX15^+/−^* and *Dkk3-Cre^+/−^;ZZEF1-ALOX15^−/−^* at P11; *HRGP-Cre^+/−^;ZZEF1-ALOX15^+/−^* and *HRGP-Cre^−/−^;ZZEF1-ALOX15^+/−^* at week 8) for immunohistochemistry to assess reporter expression and cellular morphology as previously described [32] except that retinal cryosections were incubated with tdTomato antibodies (Origene; AB8181-200, 1:400) instead of green fluorescent protein (GFP) in combination with Cone Arrestin (Millipore-Sigma; AB15282; 1:1,000) and Rhodopsin (Sigma-Aldrich; O4886, 1:10,000) [32]. All comparisons were made between transgenic mice and littermate controls lacking either *Cre* or transgene.

In addition to ocular phenotyping, total RNA was isolated from the retinae of *HRGP-Cre^+/−^;ZZEF1-ALOX15^+/−^* and *Dkk3-Cre^+/−^;ZZEF1-ALOX15^+/−^* mice (weeks 152 and 77, respectively) using the Monarch Total RNA Miniprep Kit (New England Biolabs) as per the manufacturer’s protocol. Eluates were treated with DNase I (Thermo Scientific) for 30 min at 37 °C and subsequently purified using the PuroSPIN Total RNA Purification Kit (Luna Nanotech). The resulting RNA was reverse transcribed to cDNA using the LunaScript RT SuperMix kit (New England Biolabs), with 100 ng of RNA in a 20-μL reaction. To assess transgene expression at the RNA level, PCR targeting the *ZZEF1-ALOX15* fusion junction and *Cre* (Table S2) was performed with 2× Taq FroggaMix (FroggaBio) using 5 ng of resulting cDNA from each sample. PCR products were resolved on a 2.0% agarose gel and imaged using the ChemiDoc Imaging system (Bio-Rad, Hercules, CA, USA). Bands of interest were excised, isolated by centrifugation (10,000×*g* for 10 min) and Sanger sequenced at TCAG.

### Whole-genome LRS analysis of *ZZEF1-ALOX15* transgenic mice

Genomic DNA extracted from an ear notch of a *ZZEF1-ALOX15^+/−^* transgenic mouse (F_4_ descendant of the original founder) was sequenced using the PacBio Revio at TCAG. Raw barcode-assigned HiFi reads provided in a BAM file were then mapped and analyzed for initial assessment. Reads were converted to FASTQ format using SAMtools v1.21 [35] and aligned to the mouse reference genome (GRCm39) using minimap2 v2.24 [36]. We used the “map-pb” preset in minimap2 with soft clipping enabled to retain sequences failing to map to the reference genome, ensuring that plasmid-derived sequences were not eliminated. Additional quality filtering was not applied after alignment to preserve exogenous reads in the event of substantial transgene rearrangement. The resulting BAM file was then indexed with SAMtools and analyzed using the Integrative Genomics Viewer (IGV) v2.19.6 [37]. To determine average genome-wide coverage and the number of mapped reads, mapping statistics were generated using the SAMtools “stats” command. Coverage was calculated by dividing the total bases mapped among barcode-assigned and primary mapped reads (determined from Concise Idiosyncratic Gapped Alignment Report operations) by the haploid GRCm39 genome size.

To analyze transgene structure, reads containing transgenic DNA were identified and extracted by aligning raw reads to a multi-FASTA file (Data File S1) containing independent “bait” sequences of vector components (CAG promoter, *ZZEF1*, *ALOX15*, LSL cassette, tdTomato, luciferase, and backbone plasmid elements) using minimap2 with the “map-pb” preset and soft clipping enabled. Reads extracted by more than one vector component were deduplicated by read ID. To avoid capturing endogenous orthologs, reads identified by *ZZEF1* or *ALOX15* baits were only retained if they were supported by at least one additional vector component. Resulting reads were then imported into Geneious Prime v2025.1.3 (www.geneious.com). This baiting strategy was applied separately to the barcode-assigned and -unassigned HiFi read datasets. All baited reads were manually inspected in Geneious Prime for transgene elements before downstream analysis. Here, we employed the “Annotate and Predict” tool to resolve transgene-associated elements within individual reads against a database of manually curated plasmid features, with a ≥50% similarity threshold applied to identify vector components that may have been corrupted during integration. To perform a preliminary assessment of the *ROSA26* locus, transgene-derived reads identified by this strategy were removed from the initial alignment before re-visualizing coverage across the region on IGV.

For the determination of the integration site, transgene-derived reads with terminal sequences that did not match exogenous DNA were queried against the mouse genome using BLAT [38] to identify 5′ and 3′ junctions. Any unresolved sequences that remained within these reads were further annotated using BLAST [39]. Annotated junctional reads were aligned to the candidate integration site using the minimap2 plugin within Geneious Prime. A final integration site map with partial assembly of the concatemer was then generated using a consensus sequence of mapped reads. Junction sequences were aligned with the corresponding host-genome and exogenous reference sequences to inspect repair signatures including homologies/microhomologies, local rearrangements, and insertions/templated insertions at each integration breakpoint. De novo assembly of the entire transgene-derived dataset was attempted using Flye v2.9.6 [40] and the native Geneious Prime de novo assembler. In silico analysis of potential genome-wide off-target Cas9 editing sites was performed using Cas-OFFinder [41] (≤4 mismatches). Concurrently, genome-wide structural variants (SVs) were called using pbsv v2.6.2 (https://github.com/PacificBiosciences/pbsv) across the GRCm39 reference genome, with variants passing the standard pbsv quality filter (FILTER=PASS) retained for downstream analysis. Potential editing sites were then cross-referenced with the retained SV calls to assess genome-wide Cas9-mediated rearrangements.

To assess site-level CpG methylation across the CAG promoter, raw HiFi reads identified by the CAG sequence bait were extracted using SAMtools. Resulting reads were subsequently aligned to the promoter reference and indexed using pbmm2 v26.1.0. Read-level 5-methylcytosine (5mC) calls generated by Jasmine (v2.3.0) were then parsed using pb-CpG-tools v3.0.0 (https://github.com/PacificBiosciences/pb-CpG-tools/), where CpG dinucleotides with modification scores ≥50 were classified as methylated.

Key tools, commands, and parameters utilized for the whole-genome LRS analysis are provided in Data File S3.

### Validation of off-target transgene integration

Confirmatory PCR spanning the 5′ and 3′ transgene integration junctions was performed with 2× Taq FroggaMix (FroggaBio) to verify allele status in all transgenic and control mice undergoing biochemical and *Casr* expression analysis (Table S2).

*Casr* expression in the retina was assessed using reverse transcription quantitative PCR (RT-qPCR). RNA was isolated from both retinae of a female *ZZEF1-ALOX15* mouse (*Dkk3-Cre^−/−^;ZZEF1-ALOX15^+/−^*, week 77) and a related sex-matched control (maternal first-cousin; *Dkk3-Cre^−/−^;ZZEF1-ALOX15^−/−^*, week 74). RNA from the control mouse was isolated using the RNeasy Plus Mini Kit (QIAGEN), following homogenization through a QIAshredder column according to the manufacturer’s protocol. RNA from the *Dkk3-Cre^−/−^;ZZEF1-ALOX15^+/−^* mouse was isolated as described earlier (refer to “Retinal phenotyping of Cre-activated litters” section). Resulting RNA samples were reverse transcribed using the LunaScript RT SuperMix Kit (New England Biolabs). To explore expression differences in the transgenic mouse relative to wild-type control, qPCR targeting exons 2 and 3 of *Casr* was performed using 5 ng of resulting cDNA, with each sample run in technical triplicates and normalized to *Ppib* (Table S2).

Whole blood from the saphenous vein and spot urine were collected from transgenic mice (*ZZEF1-ALOX15^+/−^*, *n* = 9 for both blood and urine) and controls lacking the transgene (*ZZEF1-ALOX15^−/−^*, *n* = 10 for blood and *n* = 8 for urine, as 2 aged mice failed to produce urine) for calcium analysis. Animals included males and females with ages ranging from 23 to 111 weeks and 30 to 122 weeks for serum and urine analysis, respectively. Whole blood was kept at 4 °C prior to centrifugation at 1,300×*g* for 15 min to isolate serum, of which a minimum of 15 μL was subsequently frozen at −80 °C until testing. A minimum of 25 μL of urine was stored at −80 °C until time of testing. Isolated serum calcium and urinary calcium/creatinine concentrations were measured using the Beckman Coulter AU480 chemistry analyzer. Boxplots and statistical analyses were generated using R v4.5.1 [42] in RStudio v2025.05.1.513 [43] with tidyverse v2.0.0 [44], ggplot2 v3.5.2 [45], and ggpubr v0.6.1 [46]. Associations between genotype and serum calcium or urinary calcium-to-creatinine ratios were analyzed using multiple linear regression models adjusted for age and sex, with genotype effect reported with 95% confidence intervals (CIs).

## Results

### Functional assays of *ZZEF1-ALOX15* mice suggest transgene silencing

Co-transfection of *pCAG-ZZEF1-ALOX15-T2A-L2T* with pCMV-Cre in HEK293T cells produced strong tdTomato reporter fluorescence after 24 h, confirming construct functionality in the presence of Cre (Fig. 1B). Mice generated using this construct (*ZZEF1-ALOX15* mice) were crossed to *HRGP-Cre* and *Dkk3-Cre* driver lines, designed to activate transgene expression in cone photoreceptors or most retinal progenitor cells, respectively. However, neither transgene activation nor a retinal phenotype was observed in *HRGP-Cre^+/−^;ZZEF1-ALOX15^+/−^* or *Dkk3-Cre^+/−^;ZZEF1-ALOX15^+/−^* mice. Retinal harvests from *HRGP-Cre^+/−^;ZZEF1-ALOX15^+/−^* and *Dkk3-Cre^+/−^;ZZEF1-ALOX15^+/−^* mice demonstrated successful Cre-lox recombination, evidenced by the excision of the LSL cassette located between the CMV early enhancer/chicken β-actin (CAG) promoter and the *ZZEF1-ALOX15* chimera (Fig. 1C). However, neither *HRGP-Cre^+/−^;ZZEF1-ALOX15^+/−^* nor *Dkk3-Cre^+/−^;ZZEF1-ALOX15^+/−^* mice exhibited outer retinal damage or lipofuscin deposition relative to controls on fundus and OCT examination, expected indicators of retinal damage (Fig. 1D to F). While one (1 of 4) transgenic mouse (*HRGP-Cre^+/−^;ZZEF1-ALOX15^+/−^*) showed mild age-related drusenoid lesions at week 54, similar findings were observed in a control littermate (*HRGP-Cre^+/−^;ZZEF1-ALOX15^−/−^*). Live imaging with a tdTomato excitation/emission filter at week 5 did not detect tdTomato fluorescence in *Dkk3-Cre^+/−^;ZZEF1-ALOX15^+/−^* mice (Fig. 1E). Also, immunohistochemistry of retinal cryosections at P11 (*Dkk3-Cre^+/−^;ZZEF1-ALOX15^+/−^*) and 8 weeks (*HRGP-Cre^+/−^;ZZEF1-ALOX15^+/−^*) similarly did not detect tdTomato signal or any morphological abnormalities (Fig. 1F). In line with this, end-point RT-PCR failed to detect the *ZZEF1-ALOX15* fusion junction transcript from retinal cDNA of either Cre-activated line, despite detection of Cre transcript (Fig. S1). Collectively, the absence of tdTomato reporter activation, retinal pathology, and RNA expression in *HRGP-Cre^+/−^;ZZEF1-ALOX15^+/−^* and *Dkk3-Cre^+/−^;ZZEF1-ALOX15^+/−^* mice is consistent with transgene silencing.

### Long-read sequencing reveals unintended concatenation and off-target integration of the transgene in the *ZZEF1-ALOX15* mouse

While genotyping targeting the tdTomato reporter of founder mice and F1 carriers confirmed germline transmission of the transgene cassette (Table S2), 3′ junction PCR (spanning the ~4.3-kb *ROSA26* homology arm and into the endogenous locus) was consistently challenging to obtain and interpret, leaving uncertainty regarding the transgene’s integration site and structural architecture. To resolve this, we sequenced the genome of a *ZZEF1-ALOX15^+/−^* mouse, obtaining 428 GB of data consisting of 7.5 million HiFi reads. Alignment to the GRCm39 reference genome achieved a mean genome-wide coverage of 49*×* (>99.99% of primary HiFi reads mapped, corresponding to 7,502,968 of 7,503,051 reads, including 11,225 low-quality alignments). Furthermore, we identified 410 vector-derived reads from raw sequencing data upon alignment to vector component sequences (including 2 reads identified from the barcode-unassigned HiFi read dataset), corresponding to 0.005% of all reads generated.

Initial visualization of alignments using IGV revealed a disproportionately high depth of reads containing plasmid elements at the intended *ROSA26* locus, inconsistent with single-copy integration (Fig. S2A). These reads mapped to this locus solely through the 2 *ROSA26* homology arms present in the plasmid. Terminal sequences of these reads did not span into the endogenous locus, and alignment upon the removal of transgene-derived reads identified through our baiting strategy was consistent with diploid sequencing coverage at the locus (50× across *ROSA26* and 48× across the intended integration site within the locus) (Fig. S2B). This pattern suggested that construct integration had likely occurred elsewhere in the genome. Bait extraction and annotation of individual reads containing plasmid elements identified extensive plasmid–plasmid junctions, consistent with concatenation (Fig. 2).

**Fig. 2.**
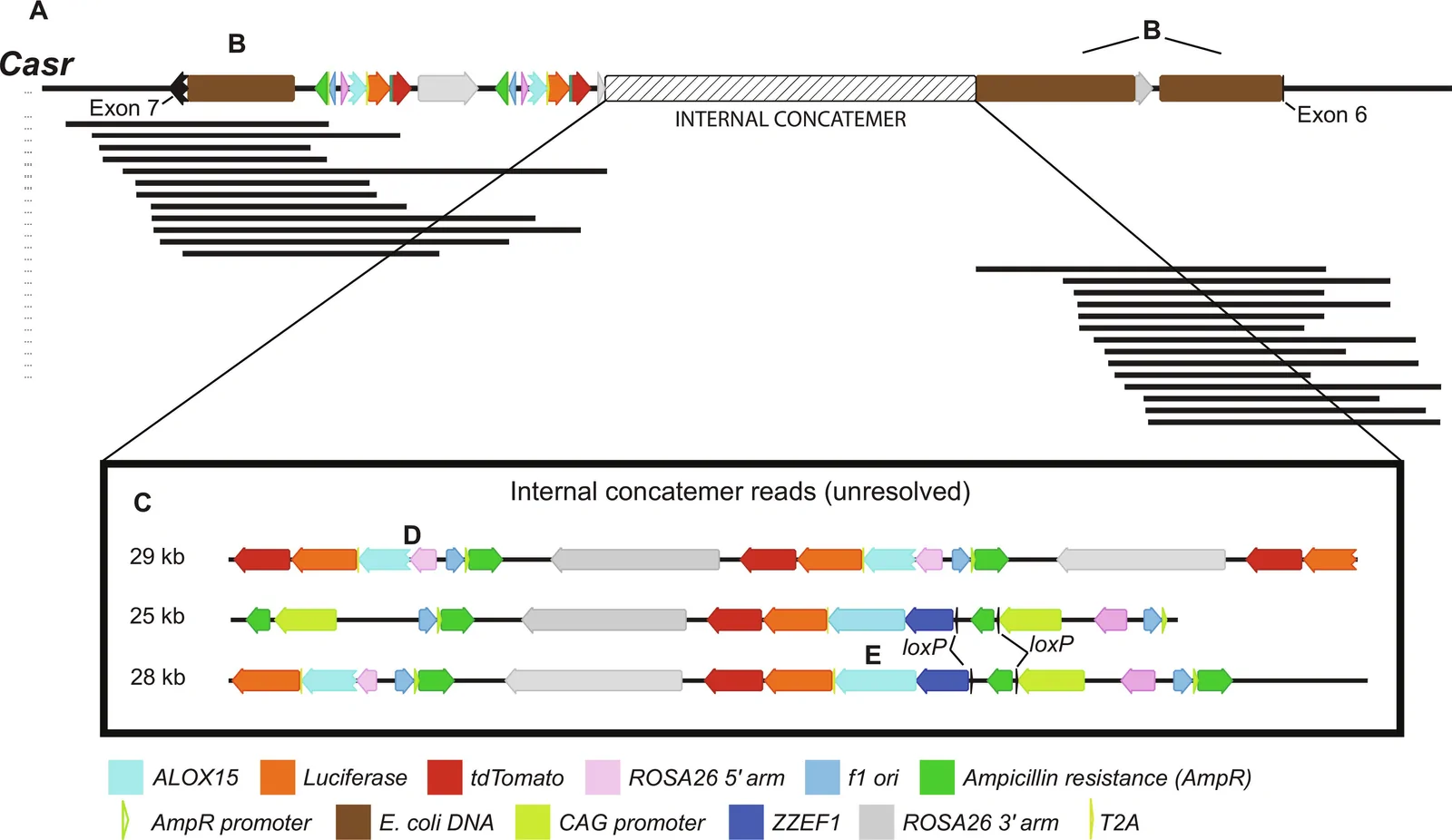
(A) Annotated consensus sequence generated using Geneious Prime from junction-spanning HiFi reads shows concatemer integration into *Casr*, accompanied by (B) *Escherichia coli* genomic DNA at both breakpoints (7.7 kb at the 3′ junction and 8.8 kb at the 5′ junction). At the 5′ end of the concatemer, the 8.8-kb *E. coli* fragment is followed by a truncated 3′ *ROSA26* homology-arm segment and an additional 11.6-kb *E. coli* fragment. A 1.8-kb deletion spans exons 6 and 7 of *Casr*. (C) Individual reads sampled from within the concatemer (not aligned) demonstrate at least 3 head-to-tail transgene-derived copies and the presence of at least one structurally preserved expression cassette. The longest read supporting the concatemer was ~29 kb. (D, representative) Multiple copies of the transgene (including at the 3′ junction) contain truncated *ZZEF1-ALOX15* fusion sequences and lack the CAG promoter. (E) At least one copy containing the structurally preserved expression cassette was identified.

Construct-derived reads with termini that did not match against a database of construct features were considered potential genome–transgene junctions and used to identify the concatemer integration site. We identified 26 junctional reads aligning to *Casr* on chromosome 16 (6.3% of all transgene-derived reads). Of these, 14 aligned to the 5′ junction and 12 to the 3′ junction of the concatemer (Fig. 2A). The longest reads obtained from the 5′ and 3′ junction were 25 and 35 kb, respectively. Additional transgene integration sites were not detected among the vector-derived reads. IGV visualization of the *Casr* locus supported a heterozygous transgene integration which was coincident with a 1.8-kb deletion (Chr16:36,316,176–36,317,973) spanning from the terminus of exon 6 toward the start of exon 7 (Fig. S3). All reads corresponding to the integrated allele supported the 1.8-kb deletion, suggesting that the deletion occurred concurrently with transgene integration. Furthermore, sequence homologies or microhomologies between the host-genome and exogenous sequences were not identified at either breakpoint nor were templated insertions or local rearrangements. Whereas the expression vector featured an LSL cassette and the *ZZEF1-ALOX15* chimera immediately downstream of the CAG promoter (Fig. 1A), the 2 head-to-tail copies captured at the 3′ integration junction displayed substantial structural disruption, evidenced by the absence of the CAG promoter and *ZZEF1* sequences (Fig. 2D). Furthermore, backbone vector elements such as the ampicillin resistance gene and a truncated *ROSA26* 5′ homology arm were discovered immediately upstream of the fusion gene, characterized by the complete loss of the *ZZEF1* sequence and partial truncation of *ALOX15*. Along with tdTomato and luciferase, this truncated fusion gene was oriented opposite to the direction of *Casr* transcription. In silico prediction using Cas-OFFinder did not identify any off-target Cas9 editing sites at the deletion site or elsewhere within the *Casr* locus.

Genome-wide SV calling with pbsv identified 28,647 quality-filtered variants (Table S3). Among these, a ~288-kb duplication on chromosome 9 (chr9:46,782,333–47,070,700) was found to overlap with a site predicted by Cas-OFFinder. However, given that the site contained 4 mismatched bases relative to the sgRNA and was located ~53 kb from the nearest breakpoint, this duplication was not considered a likely Cas9-mediated off-target event. No other predicted off-target sites were found near or within an SV.

From within the internal concatemer, the longest read obtained (29 kb) captured 3 head-to-tail-oriented copies of the transgene with structural losses like those observed at the integration junction, further highlighting the extent of the rearrangement beyond the expected 11-kb integration cassette (Fig. 2C). Among all transgene-derived reads, we identified at least one copy of the structurally preserved expression cassette (Fig. 1A), which was recovered from the internal concatemer rather than the junctional reads (Fig. 2E). De novo assembly of the complete concatemer could not produce a plausible consensus sequence beyond the integration junctions, likely because the repeat array could not be contained within the read lengths obtained. Consequently, the orientation and precise location of the preserved copy within the overall structure remains unknown.

To explore the mechanism underlying transgene silencing from the structurally preserved cassette, we used pb-CpG-tools to perform 5mC profiling across CAG promoter sequences from ear-notch DNA. Using a modification-score cutoff of ≥50, 95.7% (176/184) of CpG positions across the CAG promoter were classified as methylated, consistent with promoter hypermethylation (mean modification score, 91.4; mean coverage, 43×; Fig. 3). Specifically, 100% (20/20) of CpG positions within the CMV enhancer, 78% (25/32) in the chicken β-actin promoter, and 99.2% (131/132) in the chimeric intron were predicted to be methylated (Fig. 3). As methylation status could not be resolved in a copy-specific manner, this profiling represents the aggregate methylation state across all CAG elements present. Given a mean genome-wide coverage of 49×, the observed coverage at the CAG sequence is consistent with approximately 2 copies of the promoter present within the concatemer. We note that 12/52 CpG sites located across the CMV enhancer and chicken β-actin promoter produced modification scores between 36 and 66 (Data File S4). This may reflect differing methylation status across copies at these sites or local CpG-specific variability within copies. Nevertheless, for 87% (160/184) of CpG sites across the promoter, ≥90% of supporting reads were concordant in methylation status, suggesting a high degree of consistency between copies of the CAG promoter overall. Thus, our data indicate widespread hypermethylation across CAG promoter sequences within the concatemer. The annotated output file generated using pb-CpG-tools, reporting modification score and coverage for each CpG site, has been provided in Data File S4.

**Fig. 3.**
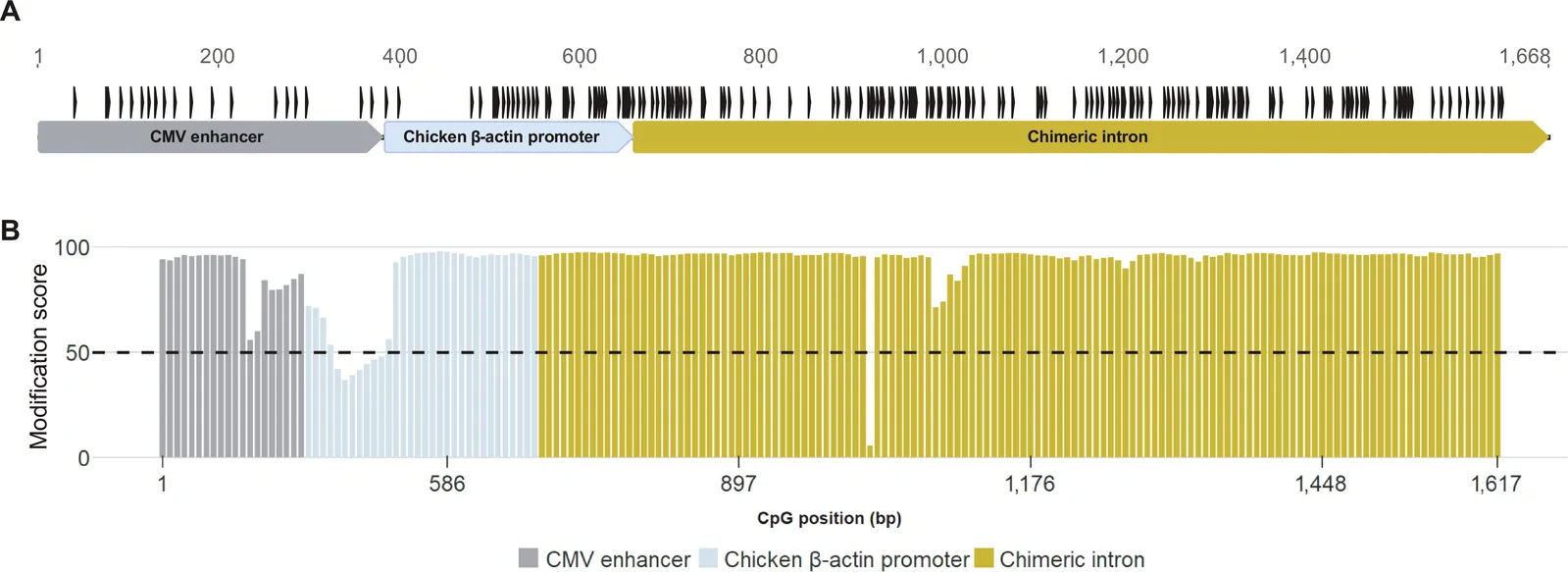
(A) Schematic showing the 3 regions of the CAG promoter in pCAG-ZZEF1-ALOX15-T2A-L2T, generated using Geneious Prime. CpG dinucleotides are indicated by black vertical bars. (B) Bar chart showing the aggregate 5-methylcytosine modification scores (1 to 100) of CpG dinucleotides across all copies of the CAG promoter present within the concatemer, indicating hypermethylation within copies of the transgene promoter. Methylation status was called from PacBio HiFi reads using Jasmine and parsed using pb-CpG-tools. Colored bars denote sites within the CMV enhancer, chicken β-actin promoter, and chimeric intron regions. The horizontal dashed line indicates the modification score threshold of 50 used to determine CpG dinucleotide methylation status.

Notably, BLAST analysis of concatemer-flanking sequences revealed co-integration with *Escherichia coli* genomic DNA contaminants (Fig. 2B)*.* At the 3′ integration junction, we identified a 7.7-kb fragment containing 5 neighboring genes from the *E. coli* genome (*pgl*, *ybhD*, *ybhH*, *ybhI*, and *ybhJ*) (Fig. S4). A second 8.8-kb fragment was detected at the 5′ junction, comprising 6 genes (*ybhA*, *modA*, *modB*, *modC*, *galE*, and *galT).* Notably, these 2 segments map to a single contiguous region of the *E. coli* DH5α genome (Fig. S5). Downstream of the fragment at the 5′ junction, we additionally observed a partial 1.3-kb remnant of the 3′ *ROSA26* homology arm followed by a distinct 11.6-kb *E. coli* DNA fragment comprising 11 genes (*acrF*, *acrE*, *acrS*, *yhdU*, *yhdJ*, *fis*, *dusB*, *prmA*, *panF*, *yhdT*, and *accC*) (Fig. S4). Thus, we identified vector concatemerization and unintended heterozygous off-target integration in *Casr* on chromosome 16, with rearrangements involving contaminating *E. coli* DNA contributing to final transgene architecture.

### Aberrant construct integration at *Casr* is associated with altered calcium homeostasis

Breakpoint PCR confirmed the heterozygous transgene integration across exons 6 and 7 of *Casr* in *ZZEF1-ALOX15* mice used for biochemical and expression analysis (Fig. S6). Exploratory qPCR analysis demonstrated an approximately 77% reduction of retinal *Casr* expression in a *ZZEF1-ALOX15^+/−^* mouse relative to a sex-matched wild-type control (*ZZEF1-ALOX15^−/−^*; Fig. S7). CaSR senses changes to extracellular calcium (Ca^2+^) concentration and plays a central role in maintaining systemic homeostasis, partly through parathyroid hormone (PTH) secretion [47]. Heterozygous *Casr* knockout mice recapitulate familial hypocalciuric hypercalcemia, a clinically benign condition characterized by modest elevations in serum [Ca^2+^] and reduced urinary [Ca^2+^] excretion [48,49]. To assess the physiological consequences of the genomic disruption at the *Casr* locus, we measured serum and urine [Ca^2+^] in a cohort of *ZZEF1-ALOX15* mice. We observed ~15% higher serum [Ca^2+^] in these mice relative to control littermates (11.17 ± 0.87 mg/dL vs. 9.70 ± 1.23 mg/dL) (Fig. 4A). After adjusting for age and sex, transgenic genotype was associated with a 1.25 mg/dL increase in serum calcium (95% CI, 0.18 to 2.32 mg/dL; *P* = 0.025). Urinary calcium excretion (normalized to creatinine) in our model (0.26 ± 0.15) was not significantly altered in comparison to control littermates (0.19 ± 0.03) after adjustment for age and sex (*β* = 0.023; 95% CI, −0.10 to 0.15; *P* = 0.70) (Fig. 4B). This finding was unchanged in a sensitivity analysis excluding the single high-value calcium-to-creatinine ratio observed in a *ZZEF1-ALOX15* mouse (*β* = 0.021; 95% CI, −0.06 to 0.10; *P* = 0.57). The age, sex, and raw calcium measurements of all animals used for biochemical analysis are provided in Data File S5.

**Fig. 4.**
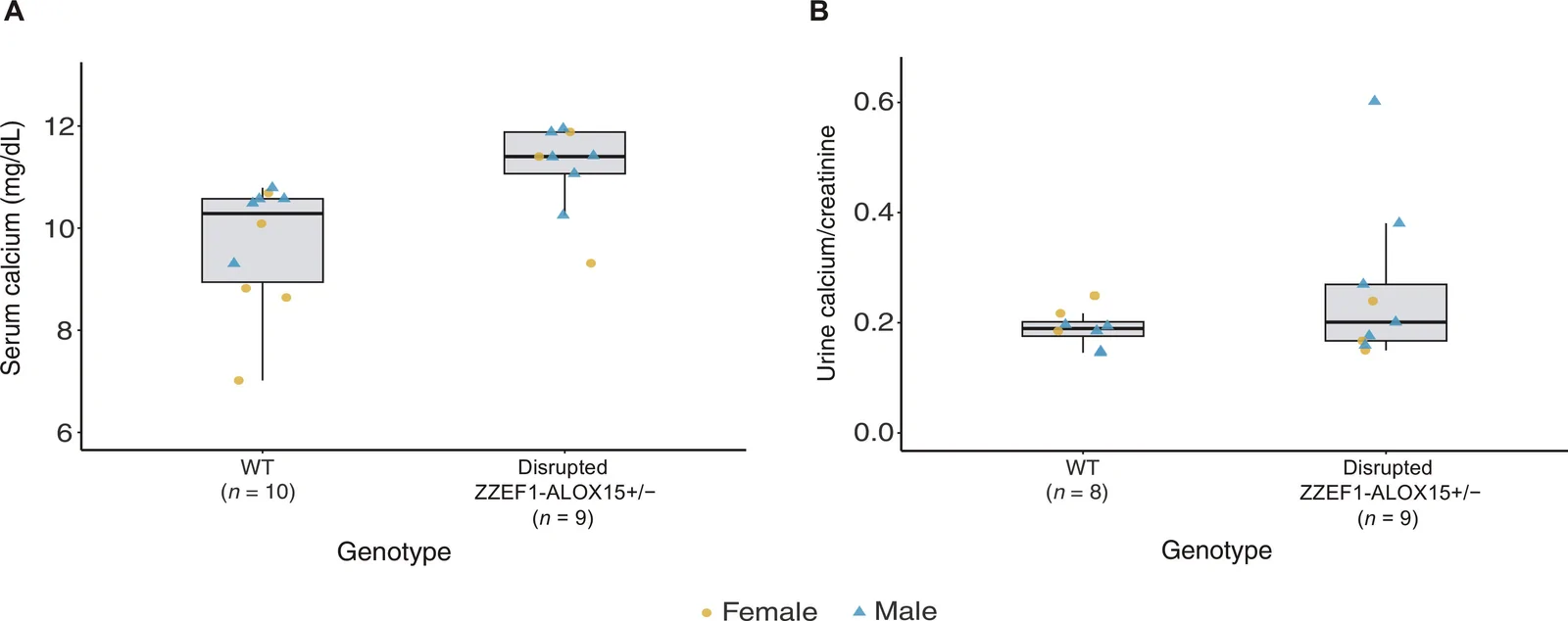
(A) Box plots of serum calcium concentration (mg/dL) in littermate controls lacking the transgene (*n* = 10) and disrupted *ZZEF1-ALOX15^+/−^* mice (*n* = 9). The disrupted line exhibited higher serum calcium levels compared with controls after adjusting for age and sex (multiple linear regression; *β* = 1.25 mg/dL; 95% CI, 0.18 to 2.32 mg/dL; *P* = 0.025). WT, wild type. (B) Box plots of urinary calcium-to-creatinine ratios (Ca/Cr) in littermate controls (*n* = 8) and disrupted *ZZEF1-ALOX15^+/−^* mice (*n* = 9). Urinary Ca/Cr in the disrupted line did not differ significantly in comparison to control mice after adjusting for age and sex (multiple linear regression; *β* = 0.023; 95% CI, −0.10 to 0.15; *P* = 0.70). Horizontal lines within each box indicate median values, 25th and 75th percentiles are denoted by the lower and upper box edges, respectively. Whiskers extend to 1.5× the interquartile range. Individual dots denote calcium measurements for each mouse and are color and shape coded by sex.

## Discussion

We identified multicopy concatenation and unintended off-target integration of the transgene into the *Casr* locus using whole-genome LRS. Consequently, aggregate 5mC profiling from ear-notch DNA indicated hypermethylation across copies of the CAG promoter within the concatemer, providing a potential epigenetic contribution for transgene silencing observed in the retina of *ZZEF1-ALOX15* mice from functional assays. Finally, biochemical analysis indicated that these rearrangements caused an unintended phenotype in the model partially consistent with that of heterozygous *Casr* knockout mice [48,50].

Indeed, we observed no evidence of *ZZEF1-ALOX15* chimera expression at the RNA level or tdTomato reporter fluorescence in the disrupted *ZZEF1-ALOX15* model, despite evidence of in vitro transgene activation from the same construct. Mouse retina previously electroporated with *ZZEF1-ALOX15* under the same promoter context (*pCAG-ZZEF1-ALOX15-IRES-GFP*) demonstrated rosette formation and thinning of the outer nuclear layer by 8 weeks, consistent with severe retinal damage [32]. In contrast, retinal lamination in these disrupted *ZZEF1-ALOX15* mice appeared unremarkable in comparison to their littermate controls, inconsistent with the expected hereditary macular dystrophy phenotype. Together, these findings implicate transgene concatenation and off-target integration in transgene silencing of the germline model.

Although the molecular basis of concatenation remains poorly understood, transgene concatemers primarily assemble through homologous recombination [51,52]. Host DNA repair mechanisms can join identical transgene copies following cell microinjection, leading to tandemly ligated and truncated expression cassettes prior to genomic integration [10]. As most reads analyzed similarly exhibited shortened transgene copies and lacked critical regulatory elements such as the CAG promoter, expression failure could be explained by structural disruption. However, the presence of at least one structurally preserved expression cassette suggests alternative silencing mechanisms. To this end, RIGS is a well-documented consequence of concatenation and is thought to occur through repressive epigenetic modifications [10]. Repetitive elements are highly regulated in mammalian cells and are typically correlated with CpG methylation [53,54]. In the context of transgenic modeling, CAG promoter-driven expression is known to be negatively correlated with methylation of CpG dinucleotides within the CMV enhancer and chicken β-actin intron [55,56]. Accordingly, CAG promoter copies within the concatemer were predominantly methylated across these regions. We therefore hypothesize that transgene silencing in the model was multifactorial, likely attributable to RIGS and truncations within the concatemer leading to the loss of the CAG promoter within some copies of the transgene.

We performed biochemical analysis in disrupted *ZZEF1-ALOX15* mice to assess the phenotypic consequences of *Casr* disruption. *Casr* encodes a dimeric G-protein-coupled receptor that is highly expressed within the parathyroid gland and the kidneys [47,57,58]. Within the parathyroid gland, the receptor exerts its calciotropic effect by repressing PTH in response to elevated serum [Ca^2+^], thereby inhibiting bone resorption and stimulating renal Ca^2+^ excretion [57]. Evidence suggests that local CaSR modulation in the kidneys occurs independently of PTH and involves down-regulation of claudins 16 and 19 [50]. Mice carrying heterozygous loss-of-function variants in *Casr* recapitulate features of familial hypocalciuric hypercalcemia, characterized by [Ca^2+^] that is modestly elevated in the blood but low in urinary excretion [48]. We found altered serum calcium homeostasis in disrupted *ZZEF1-ALOX15* mice, suggesting that transgene integration compromised CaSR function. Interestingly, despite elevated serum calcium, urinary calcium levels were not significantly different in these mice relative to controls. The phenotype observed in our disrupted model closely resembles the recently reported *Casr*^BCH002^ model, which carries an inactivating variant within the transmembrane domain of *Casr* and similarly exhibits elevated serum calcium with inappropriately normal urinary calcium excretion [50]. Although the mechanisms underlying this intermediate phenotype remain unknown, our exploratory analysis in our model demonstrated reduced *Casr* expression at the RNA level. We therefore speculate that heterozygous *Casr* disruption in *ZZEF1-ALOX15* mice may alter calcium homeostasis and contribute to an intermediate phenotype through haploinsufficiency.

Suggestive of double-strand-break repair outcomes, we identified a 1.8-kb genomic deletion at the integration site spanning from the terminus of exon 6 to the start of exon 7 in *Casr*. Junctional architecture at the integration breakpoints, devoid of homology/microhomology, local rearrangements, and templated insertions, was most consistent with non-homologous end joining. Given that in silico analysis using Cas-OFFinder did not predict Cas9 editing off-target sites in the *Casr* locus, the underlying source of the break remains unknown.

Reconstruction of junctional reads revealed the incorporation of bacterial DNA at both breakpoints. Such co-integrations have been previously reported and it is likely these rearrangements occurred with contaminating *E. coli* DNA present during model generation [26]. Notably, analysis of 40 widely used mouse lines revealed that 25% (10/40) harbored *E. coli* DNA alongside their transgenes [7]. Given that only 5% of transgenic strains published on the Mouse Genome Database have mapped integration sites, such rearrangements are considered to be more widespread than presently recognized [7].

While our study underscores the value of unbiased whole-genome LRS in characterizing complex transgene rearrangements, certain limitations warrant consideration. Cre-lox recombination, evidenced by excision of the loxP-flanked stop cassette between the CAG promoter and *ZZEF1*, was detected in both Cre-activated transgenic lines (Fig. 1C). Nonetheless, whether recombination occurred within a structurally preserved expression cassette could not be conclusively ascertained. Furthermore, while plausible off-target effects were not identified among the sites predicted by Cas-OFFinder, the possibility that some of the remaining SVs resulted from Cas9-mediated editing cannot be fully excluded due to the absence of a matched control dataset. Additionally, as CAG confers ubiquitous activity across most tissue types [59,60], the extensive hypermethylation observed across the promoter from ear-notch DNA was aberrant and strongly suggestive of transgene silencing. Nevertheless, the methylation profile obtained from this tissue may not fully reflect that of the retina. Finally, given that *ZZEF1-ALOX15^+/−^* mice were only rederived by our group, the original founder was not available for retrospective testing. Therefore, circumstances underlying the original quality-control assessment at MICAM, such as potential founder mosaicism harboring both a *ROSA26* allele and an off-target transgene concatemer, are incompletely understood.

Several strategies for evaluating transgenic mouse models have been proposed, including conventional PCR and targeted locus amplification [13,28]. However, these methods provide limited resolution when substantial deviations from transgene structure occur and may require supplementary testing, such as confirmatory sequencing or methylation analysis, to inform model viability [28]. While our analysis combined unbiased LRS with targeted transcript analysis to evaluate transgene viability, integrating whole-genome analysis with transcriptomic or proteomic profiling may provide an alternative approach for assessing the downstream consequences of transgene rearrangements during model troubleshooting or validation [8,61–63]. In our case, challenges in reconciling the technical limitations of junctional PCR with erroneous integration were evident, as routine quality control failed to detect the unintended rearrangement. As shown here and by others, LRS can facilitate comprehensive and efficient transgene integration analysis [15,64–66]. Indeed, our approach generated sufficient read and methylation data to resolve both junction sites and guide model troubleshooting, though complete reconstruction of the concatemer was not possible, as read lengths did not span the full repeat array. The choice of LRS-based mapping strategy remains largely dependent on cost, efficiency, and experiment objective, with Cas9 enrichment paired with ONT LRS currently presenting a lower-cost option with simplified downstream analysis [28,67]. Nonetheless, as recent developments in LRS technology, such as the Revio system from PacBio, continue to improve sequencing throughput while reducing per-genome costs, unbiased LRS examination is likely to become increasingly accessible, thereby enabling routine global interrogation of edited genomes. To this end, our data demonstrate the growing feasibility of unbiased whole-genome LRS as a powerful tool for the quality control of transgenic mouse models.

## Data Availability

The raw sequencing data generated in this study were deposited in the NCBI Sequence Read Archive (SRA) under BioProject accession number PRJNA1471078 and are available at the following URL: https://www.ncbi.nlm.nih.gov/bioproject/PRJNA1471078.

## References

[1] Gaj T, Gersbach CA, Barbas CF. ZFN, TALEN and CRISPR/Cas-based methods for genome engineering. Trends Biotechnol. 2013;31:397–405. 10.1016/j.tibtech.2013.04.004 PMC3694601 23664777

[2] Doudna JA, Charpentier E. The new frontier of genome engineering with CRISPR-Cas9. Science. 2014;346(6213): Article 1258096. 10.1126/science.1258096 25430774

[3] Low BE, Hosur V, Lesbirel S, Wiles MV. Efficient targeted transgenesis of large donor DNA into multiple mouse genetic backgrounds using bacteriophage Bxb1 integrase. Sci Rep. 2022;12(1):5424. 10.1038/s41598-022-09445-w PMC8971409 35361849

[4] Maino E, Wojtal D, Evagelou SL, Farheen A, Wong TW, Lindsay K, Scott O, Rizvi SZ, Hyatt E, Rok M, et al. Targeted genome editing in vivo corrects a Dmd duplication restoring wild-type dystrophin expression. EMBO Mol Med. 2021;13(5): Article e13228. 10.15252/emmm.202013228 PMC8103086 33724658

[5] Hosur V, Low BE, Wiles MV. Programmable RNA-guided large DNA transgenesis by CRISPR/Cas9 and site-specific integrase Bxb1. Front Bioeng Biotechnol. 2022;10: Article 910151. 10.3389/fbioe.2022.910151 PMC9294445 35866031

[6] Cain-Hom C, Splinter E, van Min M, Simonis M, van de Heijning M, Martinez M, Asghari V, Cox JC, Warming S. Efficient mapping of transgene integration sites and local structural changes in Cre transgenic mice using targeted locus amplification. Nucleic Acids Res. 2017;45(8): Article e62. 10.1093/nar/gkw1329 PMC5416772 28053125

[7] Goodwin LO, Splinter E, Davis TL, Urban R, He H, Braun RE, Chesler EJ, Kumar V, van Min M, Ndukum J, et al. Large-scale discovery of mouse transgenic integration sites reveals frequent structural variation and insertional mutagenesis. Genome Res. 2019;29(3):494–505. 10.1101/gr.233866.117 PMC6396414 30659012

[8] Halurkar MS, Inoue O, Singh A, Mukherjee R, Ginugu M, Ahn C, Bonatto Paese CL, Duszynski M, Brugmann SA, Lim HW, et al. The widely used Ucp1-Cre transgene elicits complex developmental and metabolic phenotypes. Nat Commun. 2025;16(1):770. 10.1038/s41467-024-54763-4 PMC11742029 39824816

[9] Hunt JM, Samson CA, Rand AD, Sheppard HM. Unintended CRISPR-Cas9 editing outcomes: A review of the detection and prevalence of structural variants generated by gene-editing in human cells. Hum Genet. 2023;142(6):705–720. 10.1007/s00439-023-02561-1 PMC10182114 37093294

[10] Smirnov A, Battulin N. Concatenation of transgenic DNA: Random or orchestrated? Genes. 2021;12(12):1969. 10.3390/genes12121969 PMC8701086 34946918

[11] Skryabin BV, Kummerfeld DM, Gubar L, Seeger B, Kaiser H, Stegemann A, Roth J, Meuth SG, Pavenstädt H, Sherwood J, et al. Pervasive head-to-tail insertions of DNA templates mask desired CRISPR-Cas9–mediated genome editing events. Sci Adv. 2020;6(7): Article eaax2941. 10.1126/sciadv.aax2941 PMC7015686 32095517

[12] Garrick D, Fiering S, Martin DI, Whitelaw E. Repeat-induced gene silencing in mammals. Nat Genet. 1998;18(1):56–59. 10.1038/ng0198-56 9425901

[13] Lintott LG, Nutter LMJ. Genetic and molecular quality control of genetically engineered mice. Methods Mol Biol. 2023;2631:53–101. 10.1007/978-1-0716-2990-1_3 36995664

[14] Sailer S, Coassin S, Lackner K, Fischer C, McNeill E, Streiter G, Kremser C, Maglione M, Green CM, Moralli D, et al. When the genome bluffs: A tandem duplication event during generation of a novel Agmo knockout mouse model fools routine genotyping. Cell Biosci. 2021;11(1):54. 10.1186/s13578-021-00566-9 PMC7962373 33726865

[15] Luqman MW, Jenjaroenpun P, Spathos J, Shingte N, Cummins M, Nimsamer P, Ittner LM, Wongsurawat T, Delerue F. Long read sequencing reveals transgene concatemerization and vector sequences integration following AAV-driven electroporation of CRISPR RNP complexes in mouse zygotes. Front Genome Ed. 2025;7: Article 1582097. 10.3389/fgeed.2025.1582097 PMC12174137 40535113

[16] Nakanishi T, Kuroiwa A, Yamada S, Isotani A, Yamashita A, Tairaka A, Hayashi T, Takagi T, Ikawa M, Matsuda Y, et al. FISH analysis of 142 EGFP transgene integration sites into the mouse genome. Genomics. 2002;80(6):564–574. 10.1006/geno.2002.7008 12504848

[17] Zhang R, Yin Y, Zhang Y, Li K, Zhu H, Gong Q, Wang J, Hu X, Li N. Molecular characterization of transgene integration by next-generation sequencing in transgenic cattle. PLoS One. 2012;7(11): Article e50348. 10.1371/journal.pone.0050348 PMC3503979 23185606

[18] Owen JR, Hennig SL, McNabb BR, Mansour TA, Smith JM, Lin JC, Young AE, Trott JF, Murray JD, Delany ME, et al. One-step generation of a targeted knock-in calf using the CRISPR-Cas9 system in bovine zygotes. BMC Genomics. 2021;22(1):118. 10.1186/s12864-021-07418-3 PMC7881600 33581720

[19] Siddique K, Wei J, Li R, Zhang D, Shi J. Identification of T-DNA insertion site and flanking sequence of a genetically modified maize event IE09S034 using next-generation sequencing technology. Mol Biotechnol. 2019;61(9):694–702. 10.1007/s12033-019-00196-0 31256331

[20] Peng C, Mei Y, Ding L, Wang X, Chen X, Wang J, Xu J. Using combined methods of genetic mapping and Nanopore-based sequencing technology to analyze the insertion positions of G10evo-EPSPS and Cry1Ab/Cry2Aj transgenes in maize. Front Plant Sci. 2021;12: Article 690951. 10.3389/fpls.2021.690951 PMC8358107 34394143

[21] Goodwin S, McPherson JD, McCombie WR. Coming of age: Ten years of next-generation sequencing technologies. Nat Rev Genet. 2016;17(6):333–351. 10.1038/nrg.2016.49 PMC10373632 27184599

[22] Logsdon GA, Vollger MR, Eichler EE. Long-read human genome sequencing and its applications. Nat Rev Genet. 2020;21(10):597–614. 10.1038/s41576-020-0236-x PMC7877196 32504078

[23] Sigurpalsdottir BD, Stefansson OA, Holley G, Beyter D, Zink F, Hardarson MÞ, Sverrisson SÞ, Kristinsdottir N, Magnusdottir DN, Magnusson OÞ, et al. A comparison of methods for detecting DNA methylation from long-read sequencing of human genomes. Genome Biol. 2024;25(1):69. 10.1186/s13059-024-03207-9 PMC10929077 38468278

[24] de Abreu AR, Ibrahim J, Lemonidis V, Mateiu L, Van Camp G, Op de Beeck K. Comparison of current methods for genome-wide DNA methylation profiling. Epigenetics Chromatin. 2025;18(1):57. 10.1186/s13072-025-00616-3 PMC12376410 40855329

[25] Promsawan K, Srichomthong C, Pongpanich M, Shotelersuk V. A comparison of DNA methylation detection between HiFi sequencing and whole genome bisulfite sequencing in monozygotic twins with Down syndrome. PLoS One. 2025;20(8): Article e0329593. 10.1371/journal.pone.0329593 PMC12324119 40763129

[26] Nicholls PK, Bellott DW, Cho T-J, Pyntikova T, Page DC. Locating and characterizing a transgene integration site by Nanopore sequencing. G3 Genes Genomes Genet. 2019;9(5):1481–1486. 10.1534/g3.119.300582 PMC6505145 30837263

[27] Suzuki O, Koura M, Uchio-Yamada K, Sasaki M. Analysis of the transgene insertion pattern in a transgenic mouse strain using long-read sequencing. Exp Anim. 2020;69(3):279–286. 10.1538/expanim.19-0118 PMC7445054 32051389

[28] Smirnov A, Makarenko M, Yunusova A. Transgene mapping in animals: What to choose? Int J Mol Sci. 2025;26(10):4705. 10.3390/ijms26104705 PMC12111812 40429848

[29] Dong Y, Li H, Zhao L, Koopman P, Zhang F, Huang JX. Genome-wide off-target analysis in CRISPR-Cas9 modified mice and their offspring. G3 (Bethesda). 2019;9(11):3645–3651. 10.1534/g3.119.400503 PMC6829146 31492696

[30] Tsai SQ, Zheng Z, Nguyen NT, Liebers M, Topkar VV, Thapar V, Wyvekens N, Khayter C, Iafrate AJ, Le LP, et al. GUIDE-seq enables genome-wide profiling of off-target cleavage by CRISPR-Cas nucleases. Nat Biotechnol. 2015;33(2):187–197. 10.1038/nbt.3117 PMC4320685 25513782

[31] Kosicki M, Tomberg K, Bradley A. Repair of double-strand breaks induced by CRISPR-Cas9 leads to large deletions and complex rearrangements. Nat Biotechnol. 2018;36(8):765–771. 10.1038/nbt.4192 PMC6390938 30010673

[32] Adele R, Hussein R, Tavares E, Ahmed K, Di Scipio M, Charish J, Liang M, Monis S, Tumber A, Chen X, et al. Autosomal-dominant macular dystrophy linked to a chromosome 17 tandem duplication. JCI Insight. 2024;9(23): Article e178768. 10.1172/jci.insight.178768 PMC11623951 39436697

[33] Le Y-Z, Ash JD, Al-Ubaidi MR, Chen Y, Ma J-X, Anderson RE. Targeted expression of Cre recombinase to cone photoreceptors in transgenic mice. Mol Vis. 2004;10:1011–1018. 15635292

[34] Sato S, Inoue T, Terada K, Matsuo I, Aizawa S, Tano Y, Fujikado T, Furukawa T. Dkk3-Cre BAC transgenic mouse line: A tool for highly efficient gene deletion in retinal progenitor cells. Genesis. 2007;45(8):502–507. 10.1002/dvg.20318 17661397

[35] Danecek P, Bonfield JK, Liddle J, Marshall J, Ohan V, Pollard MO, Whitwham A, Keane T, McCarthy SA, Davies RM, et al. Twelve years of SAMtools and BCFtools. Gigascience. 2021;10(2): Article giab008. 10.1093/gigascience/giab008 PMC7931819 33590861

[36] Li H. Minimap2: Pairwise alignment for nucleotide sequences. Bioinformatics. 2018;34(18):3094–3100. 10.1093/bioinformatics/bty191 PMC6137996 29750242

[37] Robinson JT, Thorvaldsdóttir H, Winckler W, Guttman M, Lander ES, Getz G, Mesirov JP. Integrative genomics viewer. Nat Biotechnol. 2011;29(1):24–26. 10.1038/nbt.1754 PMC3346182 21221095

[38] Kent WJ. BLAT—The BLAST-like alignment tool. *Genome Res*. 2002;12(4):656–664. 10.1101/gr.229202 PMC187518 11932250

[39] Altschul SF, Gish W, Miller W, Myers EW, Lipman DJ. Basic local alignment search tool. J Mol Biol. 1990;215(3):403–410. 10.1016/S0022-2836(05)80360-2 2231712

[40] Kolmogorov M, Yuan J, Lin Y, Pevzner PA. Assembly of long, error-prone reads using repeat graphs. Nat Biotechnol. 2019;37(5):540–546. 10.1038/s41587-019-0072-8 30936562

[41] Bae S, Park J, Kim J-S. Cas-OFFinder: A fast and versatile algorithm that searches for potential off-target sites of Cas9 RNA-guided endonucleases. Bioinformatics. 2014;30(10):1473–1475. 10.1093/bioinformatics/btu048 PMC4016707 24463181

[42] R Core Team. *R: A language and environment for statistical computing*. Vienna (Austria): R Foundation for Statistical Computing; 2025.

[43] Posit team. *RStudio: Integrated development environment for R*. Boston (MA): Posit Software, PBC; 2025.

[44] Wickham H, Averick M, Bryan J, Chang W, McGowan LD, François R, Grolemund G, Hayes A, Henry L, Hester J, et al. Welcome to the Tidyverse. J Open Source Softw. 2019;4(43):1686.

[45] Wickham H. *Ggplot2: Elegant graphics for data analysis*. New York (NY): Springer; 2016.

[46] Kassambara A. *ggpubr: ‘ggplot2’ based publication ready plots*. R package version 0.6.1. 2025. https://CRAN.R-project.org/package=ggpubr

[47] Yuan M, Ma T, Fan Z, Li J, Zhang S. The calcium-sensing receptor: A comprehensive review on its role in calcium homeostasis and therapeutic implications. Am J Transl Res. 2025;17(3):2322–2338. 10.62347/QGTS5711 PMC11982861 40226019

[48] Ho C, Conner DA, Pollak MR, Ladd DJ, Kifor O, Warren HB, Brown EM, Seidman JG, Seidman CE. A mouse model of human familial hypocalciuric hypercalcemia and neonatal severe hyperparathyroidism. Nat Genet. 1995;11(4):389–394. 10.1038/ng1295-389 7493018

[49] Kantham L, Quinn SJ, Egbuna OI, Baxi K, Butters R, Pang JL, Pollak MR, Goltzman D, Brown EM. The calcium-sensing receptor (CaSR) defends against hypercalcemia independently of its regulation of parathyroid hormone secretion. Am J Physiol Endocrinol Metab. 2009;297(4):E915–E923. 10.1152/ajpendo.00315.2009 PMC2763782 19797241

[50] Küng CJ, Daryadel A, Fuente R, Haykir B, de Angelis MH, Hernando N, Rubio-Aliaga I, Wagner CA. A novel mouse model for familial hypocalciuric hypercalcemia (FHH1) reveals PTH-dependent and independent CaSR defects. Pflugers Arch. 2024;476(5):833–845. 10.1007/s00424-024-02927-y PMC11033242 38386045

[51] Smirnov A, Fishman V, Yunusova A, Korablev A, Serova I, Skryabin BV, Rozhdestvensky TS, Battulin N. DNA barcoding reveals that injected transgenes are predominantly processed by homologous recombination in mouse zygote. Nucleic Acids Res. 2020;48(2):719–735. 10.1093/nar/gkz1085 PMC7145541 31740957

[52] Folger KR, Wong EA, Wahl G, Capecchi MR. Patterns of integration of DNA microinjected into cultured mammalian cells: Evidence for homologous recombination between injected plasmid DNA molecules. Mol Cell Biol. 1982;2(11):1372–1387. 10.1128/mcb.2.11.1372-1387.1982 PMC369942 6298598

[53] Rollins RA, Haghighi F, Edwards JR, Das R, Zhang MQ, Ju J, Bestor TH. Large-scale structure of genomic methylation patterns. Genome Res. 2006;16(2):157–163. 10.1101/gr.4362006 PMC1361710 16365381

[54] Greenberg MVC, Bourc’his D. The diverse roles of DNA methylation in mammalian development and disease. Nat Rev Mol Cell Biol. 2019;20(10):590–607. 10.1038/s41580-019-0159-6 31399642

[55] Zhou Y, Zhang T, Zhang Q-K, Jiang Y, Xu D-G, Zhang M, Shen W, Pan Q-J. Unstable expression of transgene is associated with the methylation of CAG promoter in the offspring from the same litter of homozygous transgenic mice. Mol Biol Rep. 2014;41(8):5177–5186. 10.1007/s11033-014-3385-1 24804614

[56] Yang C, Shang X, Cheng L, Yang L, Liu X, Bai C, Wei Z, Hua J, Li G. DNMT 1 maintains hypermethylation of CAG promoter specific region and prevents expression of exogenous gene in fat-1 transgenic sheep. PLoS One. 2017;12(2): Article e0171442. 10.1371/journal.pone.0171442 PMC5291418 28158319

[57] Hannan FM, Kallay E, Chang W, Brandi ML, Thakker RV. Calcium-sensing receptor in physiology and in calcitropic and non-calcitropic diseases. Nat Rev Endocrinol. 2018;15(1):33–51. 10.1038/s41574-018-0115-0 PMC6535143 30443043

[58] Hendy GN, Canaff L. Calcium-sensing receptor gene: Regulation of expression. Front Physiol. 2016;7:394. 10.3389/fphys.2016.00394 PMC5020072 27679579

[59] Chai S, Wakefield L, Norgard M, Li B, Enicks D, Marks DL, Grompe M. Strong ubiquitous micro-promoters for recombinant adeno-associated viral vectors. Mol Ther Methods Clin Dev. 2023;29:504–512. 10.1016/j.omtm.2023.05.013 PMC10241652 37287749

[60] Cox BC, Liu Z, Lagarde MMM, Zuo J. Conditional gene expression in the mouse inner ear using Cre-loxP. J Assoc Res Otolaryngol. 2012;13(3):295–322. 10.1007/s10162-012-0324-5 PMC3346893 22526732

[61] Zhou T, Cai Y, Fang F, Cai X, Fu Y. Integrated transcriptome and proteome analysis identifies keratins as key regulators of corneal scarring in a murine model. Comput Struct Biotechnol J. 2025;27:2264–2274. 10.1016/j.csbj.2025.05.042 PMC12164021 40520593

[62] Yan N, Feng H, Sun Y, Xin Y, Zhang H, Lu H, Zheng J, He C, Zuo Z, Yuan T, et al. Cytosine base editors induce off-target mutations and adverse phenotypic effects in transgenic mice. Nat Commun. 2023;14(1):1784. 10.1038/s41467-023-37508-7 PMC10063651 36997536

[63] Wang L, Zhou R, Li G, Zhang X, Li Y, Shen Y, Fang J. Multi-omics characterization of diabetic nephropathy in the db/db mouse model of type 2 diabetes. Comput Struct Biotechnol J. 2025;27:3399–3409. 10.1016/j.csbj.2025.07.037 PMC12341518 40799908

[64] Zhang H, Li R, Guo Y, Zhang Y, Zhang D, Yang L. LIFE-Seq: A universal large integrated DNA fragment enrichment sequencing strategy for deciphering the transgene integration of genetically modified organisms. Plant Biotechnol J. 2022;20(5):964–976. 10.1111/pbi.13776 PMC9055813 34990051

[65] Bryant WB, Yang A, Griffin SH, Zhang W, Rafiq AM, Han W, Deak F, Mills MK, Long X, Miano JM. CRISPR-Cas9 long-read sequencing for mapping transgenes in the mouse genome. CRISPR J. 2023;6(2):163–175. 10.1089/crispr.2022.0099 PMC10123806 37071672

[66] Yu C, Caothien R, Pham A, Tam L, Alcantar T, Bacarro N, Reyes J Jr, Jackson M, Nakao B, Roose-Girma M. ASIS-Seq: Transgene insertion site mapping by Nanopore adaptive sampling. Methods Mol Biol. 2023;2631:135–153. 10.1007/978-1-0716-2990-1_5 36995666

[67] Iyer SV, Goodwin S, McCombie WR. Leveraging the power of long reads for targeted sequencing. Genome Res. 2024;34(11):1701–1718. 10.1101/gr.279168.124 PMC11610587 39567237

